# Within-subject effects of environmental and social stressors on pre- and post-partum obesity-related biobehavioral responses in low-income Hispanic women: protocol of an intensive longitudinal study

**DOI:** 10.1186/s12889-019-6583-x

**Published:** 2019-02-28

**Authors:** Sydney G. O’Connor, Rima Habre, Theresa M. Bastain, Claudia M. Toledo-Corral, Frank D. Gilliland, Sandrah P. Eckel, Jane Cabison, Christine H. Naya, Shohreh F. Farzan, Daniel Chu, Thomas A. Chavez, Carrie V. Breton, Genevieve F. Dunton

**Affiliations:** 1 Department of Preventive Medicine, Keck School of Medicine, University of Southern California, 2001 N. Soto Street, Los Angeles, CA 90032 USA; 2 Department of Health Sciences, California State University, Northridge, 18111 Nordhoff Street, Northridge, CA 91330 USA; 3 Department of Psychology, University of Southern California, 3620 South McClintock Ave, Los Angeles, CA 90089 USA

**Keywords:** Ecological momentary assessment, Air pollution, Stress, Physical activity, Eating, Pregnancy

## Abstract

**Background:**

Disproportionately high rates of maternal overweight and obesity among the Hispanic population before, during, and after pregnancy pose serious health concerns for both mothers (e.g., preeclampsia, gestational diabetes, weight retention) and children (e.g., elevated lifelong obesity risk). A growing body of evidence implicates environmental exposures (e.g., air pollution, metals) and social stressors (e.g., poverty, violence) in contributing to obesity-related biobehavioral processes, such as physical activity, dietary intake, perceived stress, and cortisol regulation. However, current understanding of the role of environmental exposures and social stressors on obesity-related biobehavioral processes is limited by infrequent, inter-individual measurement, and lack of personal exposure monitoring.

**Methods:**

The “Maternal and Developmental Risks from Environmental and Social Stressors” (MADRES) real-time and personal sampling study examines the within-subject day-level effects of environmental and social stressors on maternal pre- and post-partum obesity-related biobehavioral responses. Among a cohort of 65 low-income, Hispanic women in urban Los Angeles, this study uses innovative personal, real-time data capture strategies (e.g., ecological momentary assessment [EMA], personal exposure monitoring, geolocation monitoring, accelerometry) to repeatedly assess obesity-related processes during the 1st and 3rd trimester, and at 4–6 months postpartum. Day-level effects of environmental exposures and social stressors on women’s physical activity, diet, perceived stress and salivary cortisol measured across repeated days will be tested using multilevel modeling.

**Discussion:**

Hispanic women of childbearing age bear a disproportionately high burden of obesity, and this population is also unduly exposed to numerous obesogenic settings. By using innovative real-time data capture strategies, the current study will uncover the daily impacts of environmental and social stressor exposures on women’s obesity-related biobehavioral responses, which over time can lead to excessive gestational weight gain, postpartum weight retention and can pose serious consequences for both mother and child. Findings from the real-time and personal sampling study will identify key mechanistic targets for policy, clinical, and programmatic interventions, with the potential for broad-reaching public health impacts.

## Background

Increasing rates of maternal overweight and obesity before, during, and after pregnancy pose serious metabolic health concerns for mothers both pre- and post-partum. Specifically, higher pre-pregnancy weight and excessive weight gain during pregnancy increase the risk of preeclampsia and gestational diabetes), [1–8] while greater postpartum weight retention leads to elevated lifelong risk of obesity [9, 10] and chronic disease [11, 12]. Further, pregnancy-related obesity rates are disproportionately high in minority and socioeconomically disadvantaged women. Specifically, among Hispanic women of childbearing age, 40% are obese [13] and 51% gain excessive weight during pregnancy [14–17]. Understanding the causes of excessive weight gain and retention among minority and low-income women is critical to reducing the disproportionate burden of disease they bear. A growing body of evidence suggests that environmental exposures (e.g., air pollution, metals) and contextual social stressors (e.g., poverty, violence) contribute to obesity and related metabolic and cardiovascular diseases [18–31]. However, there is an overall lack of research on the etiological role of environmental exposures and social stressors in obesity-related biobehavioral responses (e.g., physical activity, dietary intake, perceived stress, cortisol) during the pre- and post-partum periods.

Traditional epidemiological studies in environmental health collect repeated measures on macro-timescale (e.g., annual or semi-annual intervals) to capture changes in exposures and outcomes that occur infrequently. However, obesity-related exposures such as air pollution and stress, and biobehavioral responses such as cortisol and physical activity levels, may change at a higher frequency than can be captured by traditional longitudinal study designs and summary measures. Therefore, the current study will collect repeated measures on a finer grained timescale (e.g., daily and within-daily intervals) to better capture intra-individual (i.e., within-person) covariation in key exposures and biobehavioral responses. By doing so, it will also reduce spatial or temporal uncertainty about the actual settings that exert contextual influence on the psychological responses or behaviors under investigation, [15] which may account for inconsistencies in observed effects of the environment on health outcomes and behaviors in previous research.

**Fig. 1:**
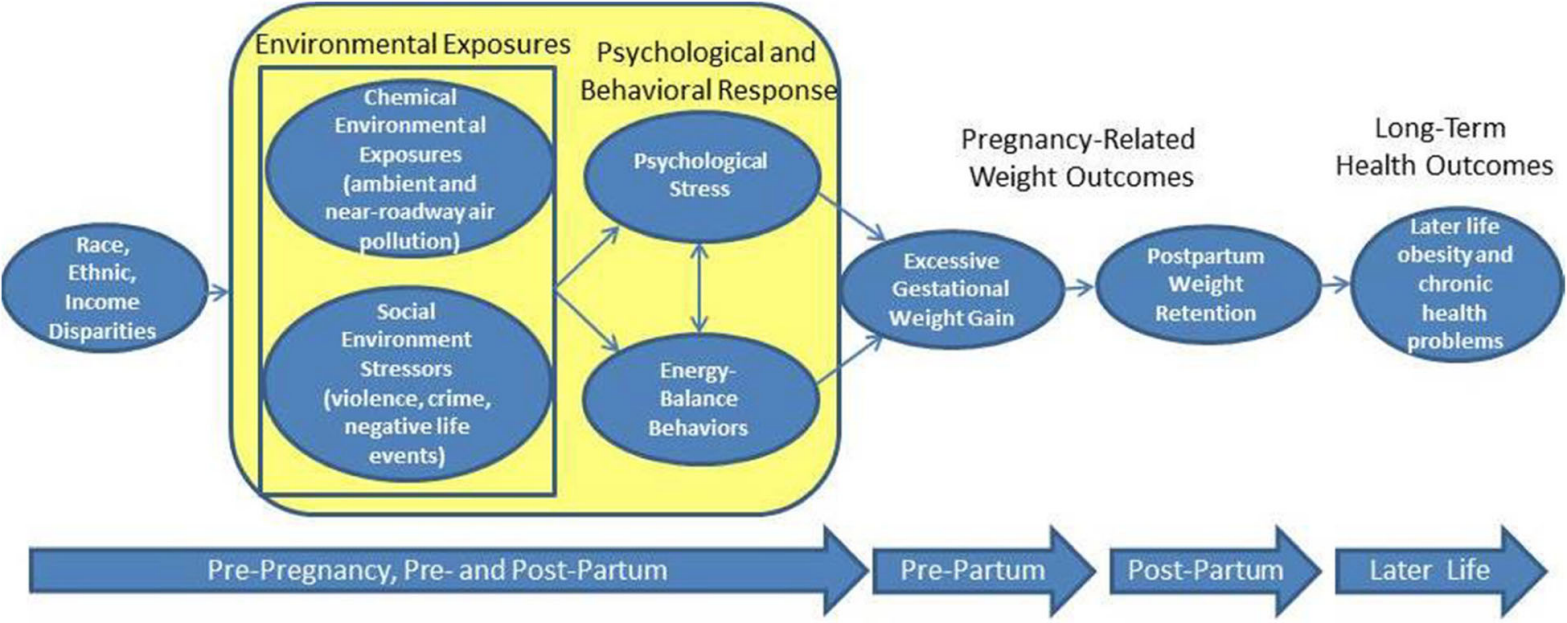
MADRES Real-Time and Personal Sampling Study Conceptual Model

To advance current investigation in this area, the “Maternal and Developmental Risks from Environmental and Social Stressors” (MADRES) real-time and personal sampling study will examine the daily effects of environmental and social stressors on maternal pre- and post-partum obesity-related biobehavioral responses. Using innovative personal, real-time data capture strategies (e.g., ecological momentary assessment [EMA], personal exposure monitoring, geolocation monitoring, accelerometry), this study will examine the day-level effects of exposures on the stress responses and energy-balance behaviors of Hispanic, low-income women during their 1st and 3rd trimester, and at 4–6 months postpartum. We hypothesize that on any given day, greater personal environmental exposures and social stressors will be associated with greater perceived stress, disrupted diurnal cortisol patterns and unhealthy patterns of dietary intake, and lower physical activity. Our conceptual model is displayed in Fig. 1. Results will identify key mechanistic targets for policy, clinical, and programmatic intervention. Given the serious long-term health consequences of excessive gestational weight gain and postpartum weight retention for both mothers and children, [3, 5, 6, 32, 33] this study has the potential for broad-reaching public health impacts.

## Methods

### Design overview

The current study uses an intensive longitudinal, observational, case-crossover design in a sample of low-income Hispanic women living in Los Angeles, California (US) during pregnancy and post-partum periods. In a case-crossover design, each woman serves as her own control to assess the within-subject effects on a repeatedly-measured dependent variable [34, 35]. A total of 65 participants are participating in four continuous days (two weekdays and two weekend days) of personal monitoring during three distinct periods: the 1st trimester, 3rd trimester, and 4–6 months post-partum. Participants are drawn from the larger MADRES prospective cohort study (*N* = 1000) focusing on mother-infant dyad health outcomes.

### Participants

The study is currently recruiting pregnant women who are receiving health care services at one of several designated healthcare facilities (e.g., community clinic, academic medical center) serving primarily low-income, Hispanic obstetric patients in urban Los Angeles. Inclusion criteria for the study are: (a) < 30 weeks since the date of mother’s last menstrual period at the time of enrollment, (b) 18 years of age or older; and (c) singleton pregnancy. Exclusion criteria are (a) HIV positive status; (b) physical, mental, or cognitive disabilities that prevent participation; or (c) current incarceration.

#### Recruitment and tracking

Recruitment takes place on a rolling basis across the first 3 years of the ongoing MADRES pregnancy cohort. MADRES cohort participants who have completed their initial cohort visit are screened for eligibility for the real-time and personal sampling study. Those who meet preliminary inclusion criteria listed above are contacted by phone and provided information on the study. If interested, an appointment is set to explain the study procedures in depth, obtain written informed consent, and provide the participant with equipment and collection materials. The longitudinal tracking and retention plan for the overall cohort includes (1) obtaining home and cell phone information from participants as well as obtaining contact information for next of kin in the event that the participant becomes unreachable, (2) sending reminders and cards and small gifts to participants to remind them of upcoming visits and congratulate them on milestones related to their new child, (3) sending quarterly newsletters to update participants on the study, and (4) annual in-person MADRES celebration events for mothers, children, and their families.

### Procedures

All study procedures are completed in either English or Spanish, by trained bilingual study staff members. At each of the three study timepoints (i.e., 1st trimester, 3rd trimester, and 4–6 months post-partum) or ‘waves’, participants complete four continuous days (two weekdays and two weekend days) of real-time assessment and continuous monitoring. Participants can complete a given study wave at any point during the window; for example, a participant is eligible to complete the 1st trimester wave at any point during her 1st trimester. Study coordinators schedule an initial at-home visit, during which the coordinator explains the study purpose and procedures, reviews the consent form, and obtains written informed consent from participants. Following informed consent, participants are trained on the use of the EMA smartphone application, the air pollution monitoring purse, and biospecimen collection kits. Following the initial visit, participants complete four days of free-living assessment taking place in the natural environment as participants conduct normal daily activities. During the assessment period, study coordinators call participants once to conduct a dietary recall assessment and once to check on compliance with the study protocol. At the end of the four-day period, study coordinators return to the participant’s home to complete the second dietary recall assessment, to collect the devices and biological samples, and to complete the wave. At each wave, several assessment modalities are used to assess within-subject variation in environmental and social exposures, and biobehavioral responses, including (a) self-report ecological momentary assessment (EMA) via smartphones, (b) body motion sensing (i.e., accelerometry), (c) personal air pollution exposure monitoring (RTI MicroPEM v3.2A), (d) location monitoring (i.e., Global Positioning Systems [GPS]), (e) ambulatory salivary cortisol assessment, (f) daily urine assessment, and (g) 24-h dietary recall on two separate days. Additional measures collected in the larger cohort study include anthropometric assessment and retrospective interview questionnaires on social, cultural, demographic, and neighborhood factors. In addition, geospatial neighborhood and census tract level social and environmental exposures are assessed based on the geocoded residential addresses.

Participants are compensated $75 in gift cards for each completed study wave; they receive $25 at the initial at-home visit, and $50 at the final at-home visit. Participants are eligible to receive an additional $80 through bonus incentives for good compliance with the study protocol, including missing fewer than five EMA surveys ($25 extra), wearing the accelerometer at least 10 h each of the four days ($25 extra), and completing each of the two 24-h dietary recalls ($15 each, up to $30 extra). The total possible compensation at each wave is $155.

### Measures

#### Ecological momentary assessment (EMA)

**Fig. 2:**
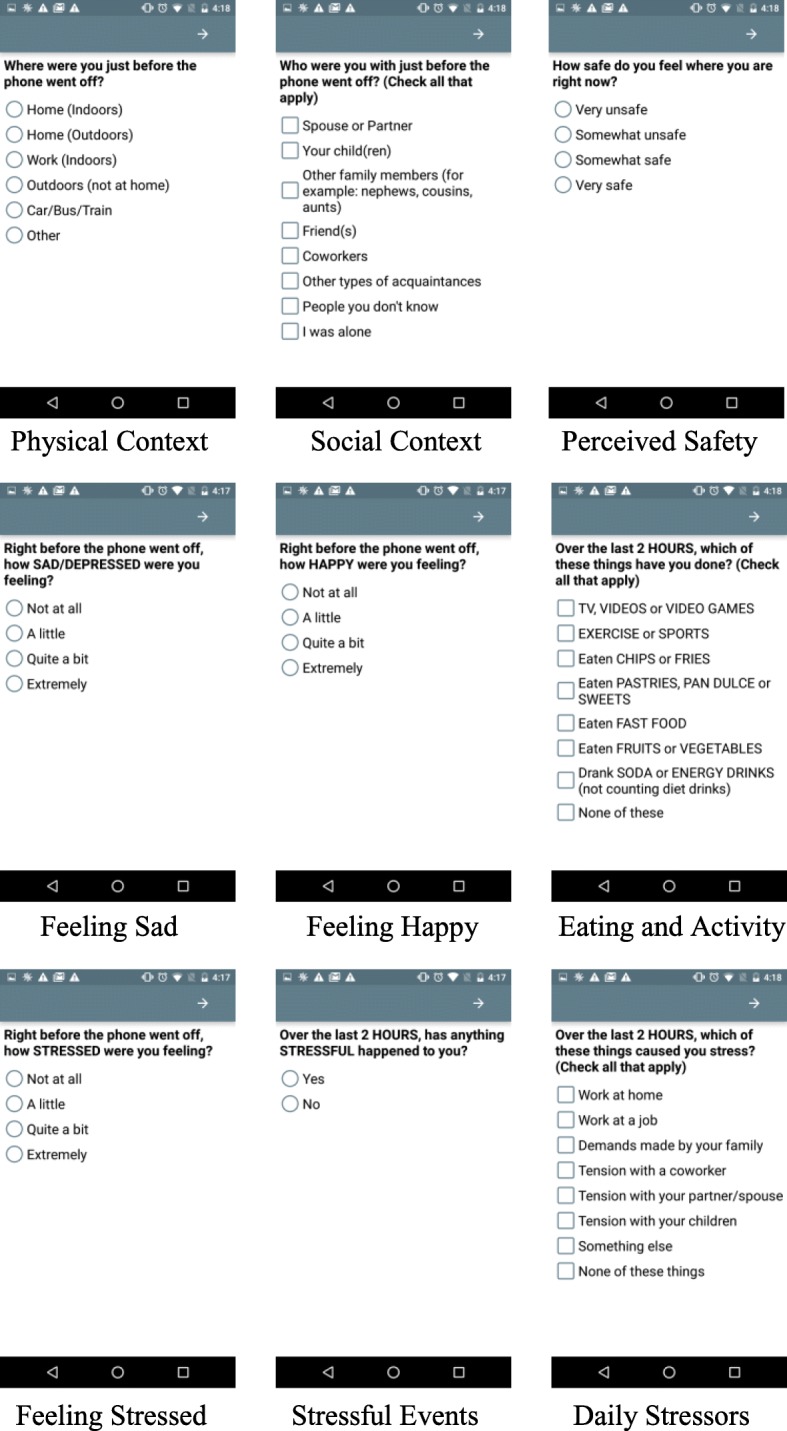
MADRES Real-Time and Personal Sampling Study Select EMA Screenshots

Real-time self-report EMA data is collected through the commercially-available MovisensXS software application “app” (https://www.movisens.com/en/products/movisensxs/) for smartphones with an Android operating system (Google USA, Inc.). EMA data from smartphones is wirelessly uploaded after each entry and stored on a cloud server, where it can be viewed by investigators to monitor compliance. Participants are provided with a Samsung MotoG phone (Model Moto G, Samsung) to use for the duration of each wave. EMA surveys are prompted at random times during pre-specified sampling windows. This was done to capture a representative sample of participants’ daily activities, while minimizing or preventing changes in participants’ current behavior in anticipation of a prompt (i.e., reactivity). Participants receive one prompt at a random time during each of the following five time-windows: wake-up – 10 am; 11 am – 1 pm; 2 pm – 4 pm; 5 pm – 7 pm, and 8 pm – bedtime. Soliciting five EMA entries per day has been shown to be acceptable for EMA studies with adults in previous work [36]. Upon hearing a signal by the app, participants are asked to complete an electronic survey on the touch screen of the phone, with each survey question displayed on a unique screen. Screenshots of select EMA items are displayed in Fig. 2.

This process requires about 2–3 min. If no entry is made, the application is programmed to emit up to two reminder signals at 3-min intervals. EMA items ask mothers to report on current affective and physical feeling states (9 items), current perceived stress (2 items) from the Perceived Stress Scale (PSS), [37] past two-hour exposure to daily stressors, including work, home, and family domains (1 item), past two-hour eating and physical activity behavior (1 item), past two-hour time use (e.g., housework, errands), current physical (1 item) and social context (e.g., home, with friends) (1 item), and rating of safety (1 item). At times when the participant is asked to carry the MicroPEM personal air pollution monitor, EMA items are also programmed to assess past two-hour compliance with wearing or carrying the monitor as well as other factors that may impact data quality and usability (4 items). In the first programmed survey of each day, participants also receive questions on the previous night’s bedtime, waketime, sleep quality, and awakenings (4 items), as well as overnight MicroPEM placement. EMA surveys are programmed in both English and Spanish, and participants select their desired language for receiving surveys at each wave. The complete list of EMA survey items is provided in Table 1.

**Table 1:** MADRES Real-Time and Personal Sampling Study EMA Items

Variable (Subscale)	Item	Response Options
Sleep Quality	1. What time did you fall asleep last night? 2. What time did you wake up this morning?	Time Input (00:00)
	How many times did you wake up during the night?	Select number
	Compared to a typical night over the past month, how well did you sleep last night?	Much worse than usual A little worse than usual About the same as usual A little better than usual Much better than usual
MicroPEM Compliance	Where did you put the air sampling bag when you were sleeping?	Next to me Same room Somewhere else
Affective and Physical Feeling States [75, 76]	Right before the phone went off, how (HAPPY, FRUSTRATED/ANGRY, STRESSED, CALM/RELAXED, SAD/DEPRESSED, TIRED, ENERGETIC, PHYSICAL PAIN, NAUSEOUS) were you feeling?	Not at all A little Quite a bit Extremely
Perceived Stress [37]	1. How certain do you feel that you can deal with all the things that you have to do RIGHT NOW? 2. How confident do you feel about your ability to handle all of the demands on you RIGHT NOW?	Not at all A little Quite a bit Extremely
Stressful Events	Since waking up this morning (Over the last 2 HOURS), has anything STRESSFUL happened to you?	Yes No
Daily Stressors [77]	Since waking up this morning (Over the last 2 HOURS) which of these things caused you stress? (check all that apply)	Work at home Work at a job Demands made by your family Tension with a coworker Tension with a spouse Tension with your children Something else None of these things
Eating and Physical Activity Behavior	Since waking up this morning (Over the last 2 HOURS), which of these things have you done? (check all that apply)	TV, VIDEOS or VIDEO GAMES EXERCISE or SPORTS Eaten CHIPS or FRIES Eaten PASTRIES, PAN DULCE or SWEETS Eaten FAST FOOD Eaten FRUITS or VEGETABLES Drank SODA or ENERGY DRINKS (not counting diet) None of these things
Time Use	Since waking up this morning (Over the last 2 HOURS), which have you done? (check all that apply)	Errands/shopping Took children to lessons/classes/activities Cooking or heating food indoors Other Vacuuming/dusting Housework/chores Work for a job Took care of an infant/toddler None of these
Physical Context [78]	Where were you just before the phone went off?	Home (Indoors) Home (Outdoors) Work (Indoors) Outdoors (not at home) Car/Bus/Train Other
Social Context [78]	Who were you with just before the phone went off? (check all that apply)	Spouse or partner Your child (ren) Other family members (for example: nephews, cousins, aunts) Friend(s) Coworkers Other types of acquaintances People you don’t know I was alone
Safety [79]	How safe do you feel where you are right now?	Very unsafe Somewhat unsafe Somewhat safe Very safe
MicroPEM Compliance	Over the past 2 HOURS, how much time did you wear the air sampling bag?	All the time Some of the time None of the time
	If you did not wear the air sampling bag sometime over the past 2 HOURS, where did you put it?	Right next to me Same room but not right next to me Somewhere else I wore it all the time
	If you were home sometime over the past 2 h, which of the following did you have (check all that apply)	Window(s) or doors open Air conditioning turned on Fan Turned on I was not home at all

#### Accelerometry

The Actigraph Inc. Model wGT3X-BT accelerometer (ActiGraph, Inc. Pensacola, FL) provides a continuous measure of body motion, at a 30-s data collection epoch. The device is attached to an adjustable belt and worn on the right hip at all times except sleeping, bathing/showering, or swimming. To reduce tilt, the belt is worn around the bottom of the pregnant stomach. Meterplus software (Santech, San Diego, CA) is used to identify periods of non-wear (i.e., > 60 continuous minutes of zero activity counts) and non-valid days (i.e., < 10 h of wear). Outcomes include daily minutes of sedentary, light, moderate, and vigorous physical activity. Cut-points for intensity levels will be consistent with studies of national surveillance data [38–40]. Waist-worn accelerometers have been shown to be acceptable and feasible across all stages of pregnancy to measure daily physical activity and sedentary time [41, 42].

#### Personal air pollution exposure monitoring

The MicroPEM v3.2A air pollution exposure monitor contains a light-scattering nephelometer (RTI International, Research Triangle, NC) to continuously measure within-day variation in personal exposure to particulate matter less than 2.5 μm in aerodynamic diameter (PM_2.5_). It is programmed to gather 60-s PM_2.5_ concentration averages continuously (20 mins on/20 mins off cycling for power optimization) across the four-day assessment period (programmed shut down at midnight of Day 4). It also collects an integrated filter-based PM_2.5_ sample for gravimetric analysis that is used to correct the nephelometer readings against known relative humidity artifacts. Based on the monitor’s motion detected using a built-in tri-axis accelerometer, wear compliance is derived. The MicroPEM is secured inside the sealed back pouch of the purse. The inlet of the MicroPEM is connected to an opaque Versilon PVC tube that is fastened along the crossbody strap of the purse with the opening beneath the shoulder in the breathing zone. Participants are asked to wear the purse during their waking hours as much as possible. At times when participants plan to stay in one location for an extended period, they are instructed to place the purse on a surface close to their body. Other modifications are suggested for times when participants are driving (i.e., remove and place on adjacent seat), showering (i.e., remove and place in nearby room away from humidity), or sleeping (i.e., remove and place on nightstand). Frequent survey questions regarding compliance with wearing the air pollution monitoring purse are built into the EMA survey questions, to guide interpretation of the data and provide key contextual information around time-activity patterns and microenvironments.

#### Location information using GPS and geospatial context using GIS

Momentary geolocation monitoring occurs continuously across the 4 days of each wave. A custom GPS application (madresGPS app) that was developed specifically for this study is installed along with the EMA application on the study smartphone. The madresGPS is an Android mobile app designed to track and record participants’ geolocations. It can record Advanced Encryption Standard (AES) geolocations in customizable intervals and resume the recording after interruptions such as the phone being turned off or running out of battery. The user interface is purposely simplified to limit accidental tampering by participants. The app separates and identifies geolocation data collected from GPS and cellular/Wi-Fi network so that the context of recording (indoor vs. outdoor) can be determined. Additional information, such as the timestamp of each entry, the number of satellites in use/view, accuracy, velocity and network connection status, is also recorded. The decryption of data from the app can be easily implemented in all sorts of platforms, such as Javascript or Java. The app is available for download from the Google Play Store (https://play.google.com/store/apps/details?id=com.wangjingke.madresgps), and its source code is publicly accessible on GitHub (https://github.com/wangjingke/madresGpsClient). Illustration and discussion on the usage of the app is also available online (https://wangjingke.com/2016/09/29/Illustration-and-discussion-of-the-MadresGPS-app). The app wakes up once every 10 s to search for and record available location data using the smartphone’s built-in location finding features (cell tower triangulation, Wi-fi networks, and GPS). Location data is saved as longitude and latitude coordinates at 10 s epochs, which will be used to develop daily activity spaces (i.e., polygon shape encompassing potential spatial-temporal exposures during a given day). Social stressors and built environmental characteristics of daily activity spaces will be abstracted from geospatial data layers using Geographic Information Systems (GIS) for the following: walkability, greenness, parks and open spaces, healthy food supermarkets and grocery stores, fast food restaurants, traffic exposure (density, proximity), crime rates and census-based demographic and socioeconomics variables (e.g., racial composition, percent below poverty, percent home ownership).

#### Salivary cortisol

Assessment of unbound serum cortisol in saliva captures the activation of the hypothalamic-pituitary-adrenal (HPA) axis in response to the physiological and psychological stress response [43]. In addition to reacting to acute stressors, cortisol concentration fluctuates in a diurnal pattern that is characterized by increased levels at awakening, a steep incline and peak at 30 min after awakening, and slow decline to bedtime. This diurnal cortisol pattern is estimated by calculating aggregated measures such as the cortisol awakening response, diurnal cortisol slope, and total concentration of cortisol over the day [44, 45]. Saliva is collected with the Salivette device (Sarstedtf, Inc., Rommelsdolf, Germany), which is a small cotton dental roll that participants are asked to gently chew and roll around in their mouths for two minutes. This strategy has been used in many daily experience studies with participant-administered collection in natural environments among pregnant and non-pregnant women [46–49]. Saliva samples are collected four times daily, immediately at awakening, 30 min post awakening, at 3:00 pm, and at bedtime for four consecutive days at each wave. The sampling times were chosen to capture the diurnal pattern of cortisol secretion [46]. The EMA app is programmed to emit auditory saliva sample reminder prompts at each of these four times to promote compliance to the sampling protocol. At the time of collection, participants directly mark on the saliva tube the date and time, and whether any eating, drinking (not water), tooth brushing, smoking or exercising had occurred in the prior 30 min. Participants are asked to store their saliva samples in their refrigerator until the end of each assessment period, at which time the samples are transferred to a research laboratory freezer for storage at − 80 °C. Samples are assayed with commercial chemiluminescence immunoassay (CLIA; IBL International, Hamburg, Germany), which has a lower detection limit of .005 μg/dL and intra- and inter-assay coefficients in the range of 3.0–4.1%.

#### Urine

Participants are provided with sample cups to give a morning urine sample on each of the four study days at each wave. Participants are instructed to collect daily ‘midstream’ samples of urine first thing in the morning after awakening, and to store the samples in a paper bag within a large plastic zip top bag in their refrigerator until the end of each assessment period. Upon receipt at the end of the study, samples are brought to room temperature and measured for specific gravity, then aliquoted and stored at − 80 °C. The urine will be analyzed for daily environmental exposures, including metals of interest (e.g. arsenic, manganese) by inductively coupled plasma mass spectrometry.

#### 24-h dietary recalls

Dietary intake data for 24-h recalls are collected and analyzed using the Automated Self-Administered 24-h Dietary Assessment Tool (ASA24®), version (2016), developed by the National Cancer Institute, Bethesda, MD. The ASA24 is a dietary assessment tool that allows for multiple 24-h recalls that are automatically coded for nutrient and food group content from the Food and Nutrient Database for Dietary Studies 2011–12 and Food Pyramid Equivalents 2011–12. For two of the four days at each assessment wave (one weekday and one weekend day), participants complete a 24-h dietary recall; the first recall is completed over the phone, and the second recall is completed in-person during the equipment pick-up visit at the end of the study wave. Although the ASA24 is designed to be self-administered, our study staff interview participants by telephone to obtain information on all eating and drinking occasions in the previous calendar day, and serve as proxy reporters, entering the dietary information directly into the ASA24 system. All study staff were trained by a single study co-investigator to ensure a standardized method of phone interviews. The decision for study staff to administer the dietary recall was made in order to accommodate participants who may not have home internet access and/or smart technology, and participants with low literacy. The ASA24 can be administered in either English or Spanish language, and has been shown to be acceptable for use in diverse multiethnic populations, including Hispanic mothers [50].

#### Anthropometric assessment

Height and weight are abstracted from mothers’ medical records during pre-natal visits. As part of the larger cohort study, mothers’ anthropometric measurements are taken in the 1st trimester, 3rd trimester, and at 12 month post-birth. Trained research coordinators measure height in duplicate to the nearest 0.1 cm using a stadiometer (Model PE-AIM-101, Perspective Enterprises, Portage, MI), weight in duplicate to the nearest 0.1 kg using a digital scale (Tanita, Perspective Enterprises, Portage, MI), and waist circumference in duplicate to the nearest 0.1 cm. Body Mass Index (BMI; kg/m^2^) is calculated according to CDC guidelines.

#### Retrospective surveys and medical record abstraction

As part of the larger parent study, participants complete a series of interviewer-administered questionnaires. Interviews were selected as an alternative to self-administered questionnaires in order to accommodate the varying levels of literacy among this population. Questionnaire measures include self-rated health status, [51] health history, household exposures and secondhand smoke, personal smoking and alcohol/drug use, medication use (e.g., corticosteroid, hormones, β-blockers), depression, [52] perceived stress, [37] stressful life events, [53] adverse childhood events, [54] pregnancy-related distress, [55] financial stress, [56] perceived neighborhood safety and social cohesion, [57] food insecurity, [58] sleep, acculturation, [59] pregnancy intendedness, pregnancy history (e.g., parity, gravidity, gestational age/preterm, delivery type, pregnancy complications), neonatal complications, postpartum distress, [60] breast feeding/infant feeding practices, [61] child care arrangement, and demographics (e.g., race/ethnicity, household income, education level, mother/father employment and occupation, hours worked per week, marital status, number and age of children, child custodial arrangement). Additionally, as part of the larger parent study, participants’ medical records are abstracted for relevant clinical measurements related to pregnancy and delivery, including participants’ height and weight, [62] while the baby’s records are abstracted throughout the first year of life.

### Data integration

The primary study aims are to understand the day-level contribution of environmental and social stressors to obesity-related biobehavioral responses. Primary predictors include daily exposure to air pollution and social stressors; primary outcomes will include daily cortisol, perceived stress, physical activity, and dietary intake. A day-level dataset will be created by linking the various sources of data by person and date. Each row in the day-level dataset corresponds to a unique person-date, and values represent the mean or average for each day; for example, all EMA stress ratings for a given day will be averaged to represent the mean for that day; and all accelerometer readings for a given day will be summarized to represent the number of minutes in each activity intensity level (e.g., sedentary, light, moderate) for that day. To examine day-level effects, environmental exposure variables (e.g., PM_2.5_ level) will be matched with proposed biobehavioral outcomes (e.g., EMA-reported stress, salivary cortisol, EMA-reported healthy and unhealthy eating, sedentary behavior, exercise/sports; accelerometer-derived physical activity) during the same day (concurrent effects) and the immediate next day (prospective effects). Additionally, a prompt-level dataset will be created by joining other data to EMA data by date or timestamp where each row corresponds to a unique person-day-prompt. The prompt-level dataset will be used for exploratory within-day analyses (i.e., how exposures at an earlier point in the day may influence biobehavioral responses later in the day).

### Statistical analyses

Initial exploratory analysis will be used to understand the distributions of all variables and to identify transformations that may better satisfy modeling assumptions, to understand correlation and possible multicollinearity of exposures, and to identify extreme observations. Throughout the modeling process, we will evaluate and modify models to ensure that inferences are not unduly influenced by erroneous modeling assumptions and/or influential observations. Data will be analyzed with SAS and R. Missing data patterns will be carefully analyzed, and the sensitivity of final results to various assumptions about missing data will be assessed [63, 64]. Specifically, we will consider imputation methods, [63, 64] adjustment for attrition propensity scores [65] based on the estimated probability of dropout for each participant, [66] and/or sensitivity analyses including only those subjects with complete follow-up. In all analyses, we will consider mother’s age, socio-economic status (SES), ethnicity, parity, pregnancy co-morbidities and complications (e.g., gestational diabetes, preeclampsia, preterm delivery), breastfeeding, and month and year (to adjust for seasonal variability in exposures) as potential confounders or effect modifiers. Other variables associated with outcomes (*p* < .10) will be included as covariates to investigate potential explanatory/confounding impact on primary hypotheses.

To quantify the daily effects of environmental exposures and social stressors (measured through personal exposure and geolocation monitoring, and EMA) on mother’s cortisol, perceived stress, physical activity and eating, we will use a multilevel modeling paradigm [67]. To illustrate the approach, we present a basic two-level model, below. Let y_ij_ denote the measured outcome (e.g. diurnal cortisol slope) for participant i at time t_ij_, where j indexes the repeated measurements. Let x_ij_ denote a temporally resolved, personal exposure metric (e.g., PM_2.5_ on a specific day) and X_i_ denote the participant-specific average of this exposure (e.g., average PM_2.5_ across all days of a given wave), permitting distinction between within-subject (level 1) and between-subject (level 2) effects [68]. Finally, let W_ij_ and Z_i_ denote a set of time-dependent (e.g., day of week) and time-independent (e.g. age, pre-pregnancy BMI) covariates, respectively,

$$ \mathrm{Level}\ 1:\kern1em \mathrm{g}\left[\mathrm{E}\left({\mathrm{y}}_{\mathrm{i}\mathrm{j}}\right)\right]={\mathrm{a}}_{\mathrm{i}}+{\mathrm{b}}_{\mathrm{i}}{\mathrm{t}}_{\mathrm{i}\mathrm{j}}+{\upbeta}_1\left({\mathrm{X}}_{\mathrm{i}\mathrm{j}}\hbox{-} {\mathrm{X}}_{\mathrm{i}}\right)+{\updelta}_1\left({\mathrm{W}}_{\mathrm{i}\mathrm{j}}\hbox{-} {\mathrm{W}}_{\mathrm{i}}\right) $$

$$ \mathrm{Level}\ 2\mathrm{a}:\kern1em {\mathrm{a}}_{\mathrm{i}}={\upbeta}_0+{\upbeta}_2{\mathrm{X}}_{\mathrm{i}}+{\updelta}_2{\mathrm{W}}_1+{\updelta}_3{\mathrm{Z}}_{\mathrm{i}}+{\mathrm{e}}_{\mathrm{i}} $$

$$ \mathrm{Level}\ 2\mathrm{b}:\kern1em {\mathrm{b}}_{\mathrm{i}}={\uptheta}_0+{\uptheta}_2{\mathrm{X}}_{\mathrm{i}}+{\updelta}_2{\mathrm{W}}_1+{\updelta}_3{\mathrm{Z}}_{\mathrm{i}}+{\mathrm{f}}_{\mathrm{i}} $$

where g(.) indicates an appropriate link function. Here, b_i_ is the daily association (random slope) of participant i’s environmental exposures or social stressors (X) with psychological stress or energy-balance behaviors (Y) over the period of observation, and a_i_ is the daily intercept of participant i’s environmental exposure or social stressor (random intercept). We fit the above two-level model simultaneously in a unified generalized linear mixed model (GLMM), [67] via standard software such as SAS or R (e.g., SAS proc. MIXED or GLIMMIX).

### Sample size estimation

The number of level-1 data points (i.e. person-days) is the unit of analysis for testing within-person (daily) effects, assuming non-randomly varying slopes. Conservatively estimating 25% missing data due to noncompliance and attrition, the level-1 sample size is 585 person-days (75% of 65 participants × 4 days × 3 waves), which will have power (1-β) = .80 to detect small effects (R^2^ = 0.013 or 1.3%) in simple linear regression. This translates to 80% power to detect a regression coefficient of 0.116 per unit change in a time-varying (daily) standard normal predictor with an error variance of 1 in a simple linear regression. Based on a simulation study, if the intra-class correlation coefficient (ICC) of the continuous response was 0.5 (rather than 0) but all other parameters remained the same and the model was fitted with a random intercept, the design has 80% power to detect a slightly larger regression coefficient of 0.124.

### Pilot study

Prior to the start of the MADRES personal and real-time sampling study, a pilot study of *n* = 12 low-income Hispanic women with young infants (M_age_ = 4.6 months, SD_age_ = 1.8 months) was conducted to gauge the acceptability and perceived burden of study devices and procedures. Each pilot participant completed a single four-day assessment period across several consecutive pilot ‘phases,’ in which new study devices and tasks were incrementally added to monitor for participant burden and acceptability, and to solicit feedback on the user experience with the goal of improving future iterations. In Phase 1, participants (*n* = 2) were asked to use EMA, wear the accelerometer, collect four saliva samples each day, and were measured for height and weight; in Phase 2, (*n* = 2) we introduced the MicroPEM air pollution exposure device; in Phase 3, participants (*n* = 4) were also asked to collect once-daily urine samples; Phase 4 (*n* = 2) was marked by the addition of a single ASA24 dietary recall, conducted in-person at the pick-up visit; and in Phase 5 (*n* = 2), a series of questions regarding MicroPEM wear compliance, time-activity and microenvironmental exposures were added to the within-day EMA surveys to guide interpretation of the air pollution exposure data. Following the four-day period, pilot participants completed a qualitative semi-structured interview and quantitative survey to gain insight into the user experience and potentially modifiable issues. Interviews revealed overall acceptance of the EMA application and surveys, though some pilot participants reported minor technical issues with the use of the study smartphone such as difficulty hearing the auditory tones (*n* = 1), difficulty with using the mobile phone provided (*n* = 2) and/or with the MovisensXS study application (*n* = 2), though overall (*n* = 7) participant reported minimal to no interruption of daily activities due to the EMA surveys. A few participants (*n* = 3) reported increased awareness of their own stress experience, behaviors, or parenting practices and beliefs because of participation in the study, and the majority of women (*n* = 7; 58.3%) stated that they would be willing to participate in a similar study in the future. Participant feedback led to a number of changes to study procedures to improve the participant experience; for example, participant comments regarding discomfort of storing urine containers within a clear plastic zip bag in their home refrigerators led us to include brown paper bags to hold each individual sample before placing into the plastic zip bag, which was adapted for use in subsequent participants. Additionally, feedback regarding difficulty of filling out the salivary cortisol labels, which ask participants to indicate collection time among and contextual information, led to modifications for improved readability and usability. Preliminary pilot data indicate that the protocol is feasible and not overly burdensome for participants.

### Ethics and data security

The protocol for the MADRES real-time and personal sampling study was approved by the Institutional Review Board at the University of Southern California (HS-15-00507). Written informed consent is secured from all participants by trained study coordinators. All study data are coded and stored on a secure password-protected server, accessible only to study staff members and approved individuals. Any individuals seeking access to coded study data are required to submit a formal request and agree to adhere to data security measures.

## Discussion

Existing studies examining associations between environmental exposures and obesity-related outcomes among pregnant women have used infrequent assessment intervals, which may obscure relationships between phenomena that vary with high frequency such as air pollution, stress, and biobehavioral responses. To address these limitations, the MADRES real-time and personal sampling study uses innovative intensive longitudinal design and mHealth strategies to examine within-subject day-level effects of environmental and social stressors on obesity-related biobehavioral responses during the perinatal period. Hispanic women of childbearing age bear a disproportionately high burden of obesity. This population is also unduly exposed to numerous obesogenic settings: lack of healthy food options and access to fruits and vegetables; [69] fewer safe parks and green spaces in which to be active; [70] elevated stressful life experiences, [71, 72] and work and home neighborhoods that are highly impacted by environmental, industrial, and other pollutants [73, 74]. The importance of the current study lies in its ability to understand how these environmental stressors and exposures can ‘get under the skin’, and lead to serious consequences for the health and well-being of pre- and post-partum women and their children. This study investigates pregnancy-related weight outcomes in a cohort of low-income and minority Hispanic women living in urban Los Angeles—a region with some of the highest air pollution levels and greatest health disparities in the U.S. By using innovative real-time data capture strategies including EMA, accelerometry, personal exposure and geolocation monitoring, the current study will uncover the daily impacts of the environmental and social stressor exposures on women’s obesity-related biobehavioral responses (e.g., elevated salivary cortisol; low levels of physical activity). Information from this study will allow us to evaluate putative associations between environmental exposures and pregnancy-related weight outcomes—a critical area that has been largely overlooked in the empirical literature. Additionally, the study design allows us to uncover the cumulative, interactive, synergistic, mediating, and day-level effects in order to uncover key mechanistic targets for policy, clinical, and programmatic intervention. Given the serious long-term health consequences of excessive gestational weight gain and postpartum weight retention for both mothers and children, results could have far-reaching public health and policy impacts, by identifying key mechanistic targets for policy, clinical, and programmatic interventions.

## Abbreviations

AES: Advanced Encryption Standard
ASA24: Automated Self-Administered 24-h Dietary Assessment
BMI: Body Mass Index
CDC: Centers for Disease Control
EMA: Ecological Momentary Assessment
GIS: Geographic Information Systems
GPS: Global Positioning Systems
HPA: Hypothalamic-pituitary-adrenal
ICC: Intra-class correlation coefficient
MADRES: Maternal and Developmental Risks from Environmental and Social Stressors
PM_2.5_: Particulate matter < 2.5 μm in aerodynamic diameter
PSS: Perceived Stress Scale
SES: Socio-economic status
